# Non-insulin-dependent insulin resistance surrogate indexes for predicting chronic kidney disease in diabetic individuals: a prospective cohort study

**DOI:** 10.1186/s12902-026-02362-3

**Published:** 2026-06-26

**Authors:** Xiaodie Mu, Hairong Li, Ting Huang, He Li

**Affiliations:** Department of Nephrology, The Second People’s Hospital of Hefei, Hefei, 230011 China

**Keywords:** Diabetes, Insulin resistance, Chronic kidney disease, Non-insulin-dependent IR surrogate index

## Abstract

**Background:**

Insulin resistance (IR) constitutes a significant pathological mechanism in chronic kidney disease (CKD) progression among individuals with diabetes. This study investigated the association between six non-insulin-dependent IR surrogate indexes and CKD risk in diabetic patients and evaluated their predictive performance.

**Materials and methods:**

Using data from the China Health and Retirement Longitudinal Study (CHARLS), multivariate Cox regression and nonlinear relationship analysis were employed to assess the association between non-insulin-dependent IR surrogate markers and CKD risk in diabetic population. Additionally, we assessed the predictive ability of each IR index by calculating the area under the curve (AUC), continuous net reclassification improvement (cNRI), and integrated discrimination improvement (IDI).

**Results:**

In total, 3,403 eligible participants were enrolled. Multivariate Cox analysis and Kaplan-Meier survival analysis demonstrated that CKD risk in the diabetic population increased with elevated of all IR indexes except eGDR. After adjusting for multiple confounders, estimated glucose disposal rate (eGDR) as a continuous variable revealed an inverse association with CKD risk [HR (95% CI): 0.914 (0.876, 0.954)]. Similarly, the highest eGDR quartile (Q4) maintained a significant protective association with CKD [HR (95% CI): 0.644 (0.488, 0.849] when analyzed by quartiles. All indexes demonstrated modest predictive performance, with eGDR showed the highest AUC, cNRI, and IDI.

**Conclusions:**

All IR surrogate indexes demonstrated significant associations with CKD risk in diabetic populations. The eGDR showed relatively better performance for CKD risk assessment, although the overall discriminative ability of all markers was modest in this population.

**Graphical Abstract:**

**Figure:**
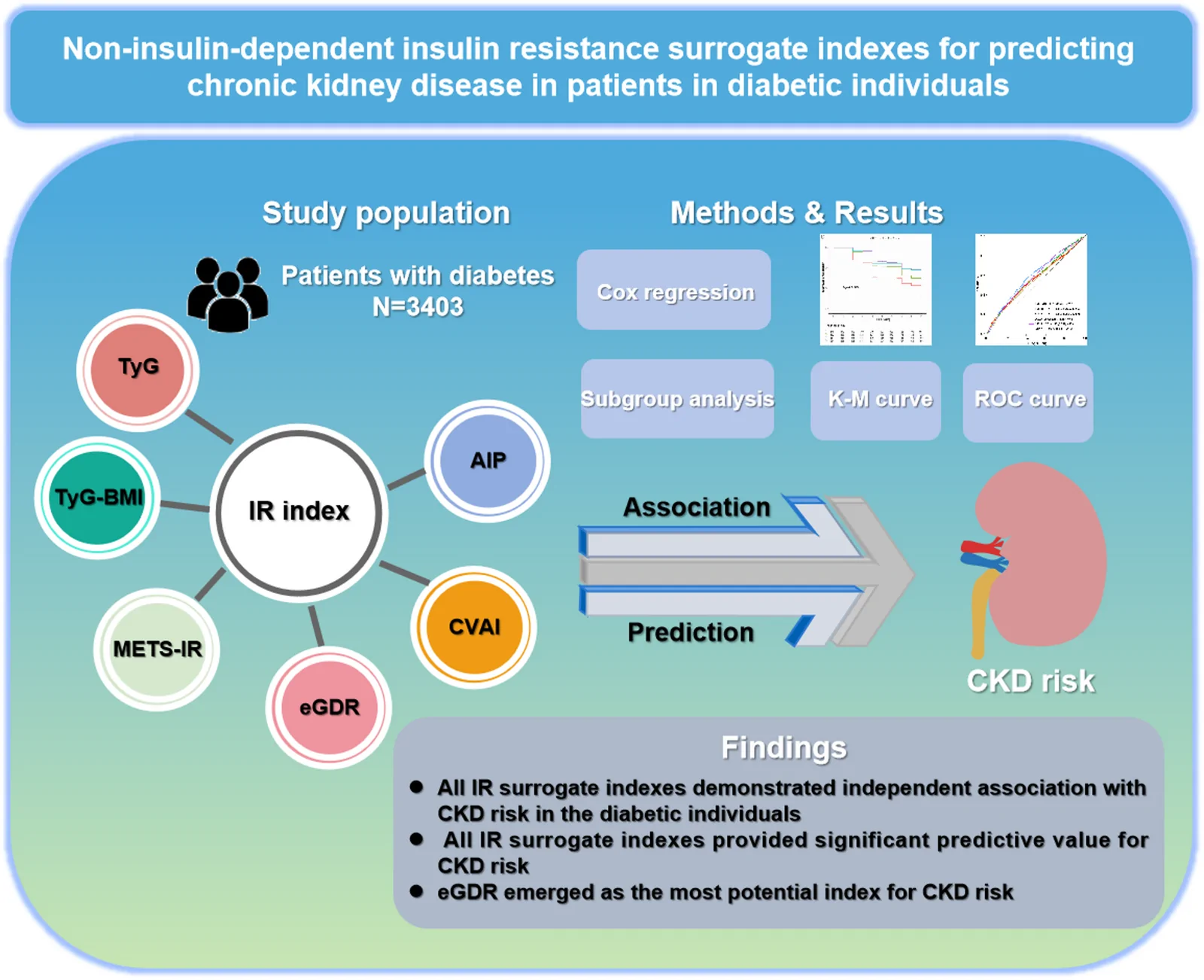

**Supplementary Information:**

The online version contains supplementary material available at 10.1186/s12902-026-02362-3.

## Introduction

Chronic kidney disease (CKD) impacts an alarming 10% of the global population [1] and ranks as the fourth fastest-rising cause of mortality [2]. This silent epidemic has become a critical public health crisis, with China bearing a disproportionately high burden [3]. Glucose metabolism dysregulation critically contributes to CKD pathogenesis [2], mediating renal damage through multiple mechanisms including disrupted cell energetics and signaling pathways [4]. Clinical evidence demonstrates that individuals with diabetes have approximately three-fold higher CKD risk than non-diabetic individuals [5], a phenomenon largely driven by insulin resistance (IR) [6]. IR contributes to both glucose metabolism dysregulation and chronic inflammatory activation, consequently raising the prevalence of cardiorenal and cerebrovascular events among individuals with diabetes [7]. Although the hyperinsulinemic-euglycemic clamp is considered the gold standard for assessing IR, its application in clinical practice and population studies is limited due to its complex protocol, invasive nature, and high cost [8]. Consequently, non-insulin-dependent alternative IR markers have been devised from basic anthropometric measures, triglyceride (TG) levels, high-density lipoprotein cholesterol (HDL-C), fasting plasma glucose (FPG) and hemoglobin A1c (HbA1c) [9], such as the triglyceride-glucose (TyG) index and its body mass index [TyG body mass index, (TyG-BMI)], along with the metabolic score for insulin resistance (METS-IR), estimated glucose disposal rate (eGDR), Chinese visceral adiposity index (CVAI), and atherogenic index of plasma (AIP). These markers have proven effective in assessing IR and exhibit robust associations with cardiovascular, cerebrovascular, renal, and metabolic diseases, thereby serving as valuable tools for risk stratification and prognosis assessment [9, 10].

Although several studies have examined the correlations between IR alternative markers and kidney disease progression, the predictive utility of these markers for CKD risk remains inconclusive. Notably, the specific relationships of these IR indexes with CKD risk in diabetics populations have not been systematically established. Importantly, no prior studies have comprehensively compared the predictive performance of these IR surrogate markers for CKD risk in diabetic populations. Thus, we performed an assessment and comparison of non-insulin-dependent IR surrogates to examine their correlation with CKD risk and compare their predictive capacity for CKD risk, focusing on Chinese diabetic population.

## Methods

### Study design

This study used data from the China Health and Retirement Longitudinal Study (CHARLS), a nationally representative prospective cohort of Chinese residents aged ≥ 45 years and their spouses [11]. The baseline survey (2011–2012) enrolled 17,708 participants through multistage probability sampling across 28 provinces, with biennial follow-ups thereafter.

**Fig. 1:**
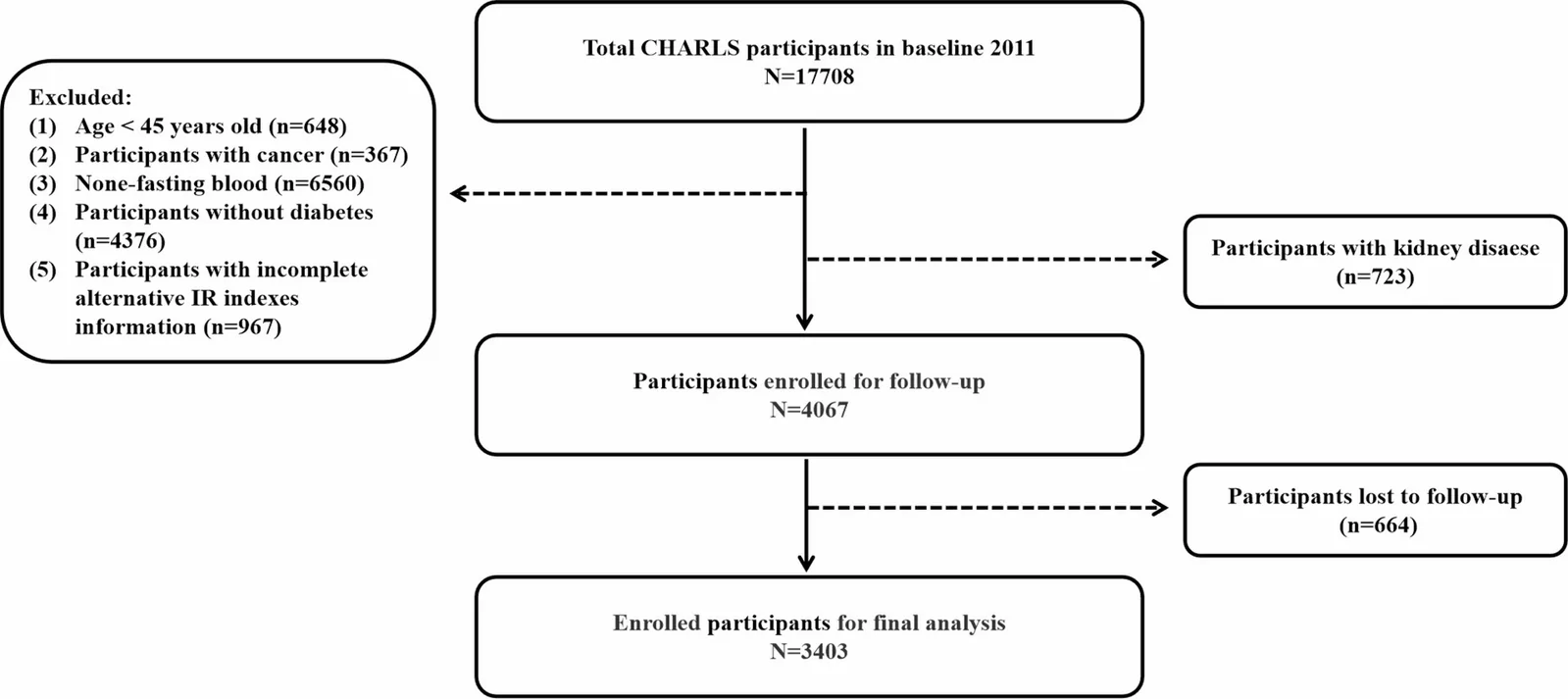
Flow chart of participants. Notes: CHARLS, China Health and Retirement Longitudinal Study; IR, insulin resistance

Using the Wave 1 baseline survey, we established the analytical cohort through sequential exclusions of participants with: (1) age < 45 years; (2) a cancer diagnosis; (3) absence of diabetes; (4) non-fasting blood samples; (5) missing data for IR surrogate markers; (6) pre-existing kidney disease; (7) missing follow-up data for kidney disease. Ultimately, the final analysis comprised 3,403 eligible individuals (Fig. 1).

### Characteristics collection and definition

The CHARLS study employed trained interviewers to collect comprehensive data through standardized protocols, including: (1) demographic characteristics (age, sex, educational level, marital status); (2) health history (smoking status, alcohol status, medical history); (3) body measurements [body mass index (BMI), waist circumference (WC), and blood pressure]; (4) hematologic and biochemical analyses.

Smoking status was defined as: (1) never smoker: participants who reported never having used any tobacco product, (2) former smoker: participants who had used tobacco products but had totally quit by the time of the survey, (3) current smoker: participants who reported currently using tobacco products, regardless of frequency or quantity.

Drinking status was defined as: (1) never drinker: participants who reported never having consumed alcoholic beverages in their lifetime, (2) former drinker: participants who had consumed alcoholic beverages in the past but reported no alcohol consumption during the past year, (3) current drinker: participants who reported any alcohol consumption during the past year, regardless of frequency.

Hypertension diagnosis was based on meeting any of the following criteria: (1) self-reported physician diagnosis, (2) current use of anti-hypertensive medications, (3) systolic/diastolic blood pressure ≥ 140/90 mmHg based on the average of three consecutive measurements [12].

Diabetes diagnosis was based on meeting any of the following criteria: (1) self-reported physician diagnosis, (2) current use of hypoglycemic medications, (3) FPG ≥ 126 mg/dL, (4) HbA1c ≥ 6.5% [13].

### Non-insulin dependent IR surrogate indexes

Each IR index was computed with these formulas as follows [14–19]:

  

$${\rm{TyG = Ln }}\left[ {{\rm{TG }}\left( {{\rm{mg/dL}}} \right){\rm{ \times FPG }}\left( {{\rm{mg/dL}}} \right){\rm{ \div 2}}} \right]$$

$${\rm{TyG - BMI = TyG \times BMI }}\left( {{\rm{kg/}}{{\rm{m}}^{\rm{2}}}} \right)$$

$$\eqalign{ & {\rm{METS - IR }} \cr & {\rm{ = Ln }}\left[ {\left( {{\rm{2 \times FPG }}\left( {{\rm{mg/dL}}} \right)} \right){\rm{ + TG }}\left( {{\rm{mg/dL}}} \right)} \right]{\rm{ }} \cr & {\rm{ \times BMI }}\left( {{\rm{kg/}}{{\rm{m}}^{\rm{2}}}} \right){\rm{ \div Ln }}\left[ {{\rm{HDL - C }}\left( {{\rm{mg/dL}}} \right)} \right] \cr}$$

$$\eqalign{ & {\rm{eGDR = 21}}{\rm{.158 - 0}}{\rm{.09 \times WC }}\left( {{\rm{cm}}} \right) \cr & {\rm{ - 3}}{\rm{.407 \times hypertension }}\left( {{\rm{yes}}\,{\rm{1}}\,{\rm{or}}\,{\rm{no}}\,{\rm{0}}} \right){\rm{ }} \cr & {\rm{ - 0}}{\rm{.551 \times HbA1c }}\left( {\rm{\% }} \right) \cr}$$

$$\eqalign{ & {\rm{CVAI }}\left( {{\rm{male}}} \right){\rm{ = - 267}}{\rm{.93 + 0}}{\rm{.68 }} \cr & {\rm{ \times age }}\left( {{\rm{years}}} \right){\rm{ + 0}}{\rm{.03 \times BMI }}\left( {{\rm{kg/}}{{\rm{m}}^{\rm{2}}}} \right) \cr & {\rm{ + 4}}{\rm{.00 \times WC }}\left( {{\rm{cm}}} \right){\rm{ + 22}}{\rm{.00 }} \cr & {\rm{ \times lo}}{{\rm{g}}_{{\rm{10}}}}\left[ {{\rm{TG }}\left( {{\rm{mmol/L}}} \right)} \right]{\rm{ }} \cr & {\rm{ - 16}}{\rm{.32 \times HDL - C }}\left( {{\rm{mmol/L}}} \right) \cr} $$

$$\eqalign{ & {\rm{CVAI }}\left( {{\rm{female}}} \right){\rm{ = - 187}}{\rm{.32 + 1}}{\rm{.71 }} \cr & {\rm{ \times age }}\left( {{\rm{years}}} \right){\rm{ + 4}}{\rm{.23 \times BMI }}\left( {{\rm{kg/}}{{\rm{m}}^{\rm{2}}}} \right){\rm{ }} \cr & {\rm{ + 1}}{\rm{.12 \times WC }}\left( {{\rm{cm}}} \right){\rm{ + 39}}{\rm{.76 }} \cr & {\rm{ \times lo}}{{\rm{g}}_{{\rm{10}}}}\left[ {{\rm{TG }}\left( {{\rm{mmol/L}}} \right)} \right]{\rm{ }} \cr & {\rm{ - 11}}{\rm{.66 \times HDL - C }}\left( {{\rm{mmol/L}}} \right) \cr} $$

  

$${\rm{AIP = log }}\left[ {{\rm{TG }}\left( {{\rm{mg/dL}}} \right){\rm{ / HDL - C }}\left( {{\rm{mg/dL}}} \right)} \right]$$

### Endpoint event

The primary CKD outcome was determined by either: (1) participant-reported physician diagnosis, (2) estimated glomerular filtration rate (eGFR) < 60 mL/min/1.73 m² (CKD-EPI formula calculated) [20]. Due to unavailable urinary albumin data, this definition primarily captures moderate-to-advanced CKD stages (G3-G5).

All participants were enrolled in the registry starting from the index date (ranging from 2011 to 2020) and were followed until the first diagnosis of any renal disease or until the outcome in 2020, whichever occurred first. Given the unavailability of precise onset times for CKD in all subjects, the endpoint event timing was determined based on established criteria from prior literature [21]. The calculation of CKD endpoint was based on two scenarios: (1) for participants without an endpoint event during follow-up: if follow-up data were available, the endpoint event time = last survey date − baseline survey date; if follow-up data were unavailable, the endpoint event time = last survey integer year − baseline survey date. (2) for participants with an endpoint event during follow-up: if follow-up data were available, the endpoint event time = (time of endpoint event wave − time of interval time)/2+ (interval wave date − baseline survey date); if follow-up data were unavailable, the endpoint event time = (integer year of endpoint event wave/2 + time of interval time/2) + (interval wave date − baseline survey date).

### Missing data processing

Multiple imputation techniques were employed to compensate for missing baseline data of non-critical variables during the final analysis. Data were missing for education level (*n* = 1, 0.3%), heart diseases (*n* = 7, 2.1%), stroke diseases (*n* = 5, 1.5%), systolic blood pressure (SBP) (*n* = 20, 5.9%), diastolic blood pressure (DBP) (*n* = 21, 6.2%), white blood cell (WBC) count (*n* = 34, 10.0%), blood platelet count (*n* = 34, 10.0%), hemoglobin (Hb) (*n* = 35, 10.3%), and blood urea nitrogen (BUN) (*n* = 1, 0.3%). Participants with missing data for critical variables or renal disease outcomes were excluded from the analysis. Sensitivity analyses were conducted to assess potential selection bias by comparing baseline characteristics among the final analytic cohort, participants with imputed data for non-critical variables, and all excluded participants.

### Statistical analysis

The variables were expressed as mean with standard deviation (SD), median (interquartile distance) and percentage. Comparative analyses between groups were executed utilizing: t-tests for normal distributions, Mann-Whitney U tests for skewed data distributions, and chi-squared tests for categorical variable comparisons. We employed multivariate Cox proportional hazard modeling to derive adjusted hazard ratio (HR), and Kaplan-Meier (K-M) survival curve estimation accompanied by log-rank statistical comparisons for assessing cumulative CKD risk. All IR indexes underwent quartile stratification (Q1 as referent). Three sequential multivariable models were specified: a crude univariate model (Model 1), a demographically adjusted model incorporating age and sex (Model 2), and a comprehensively adjusted model including additional covariates demonstrating univariate significance (*P* < 0.05) or established clinical relevance (Model 3), with multicollinearity performed via variance inflation factors (VIF) (all VIFs < 2). Nonlinear relationships were examined using restricted cubic spline (RSC) (5 knots) and threshold effect analysis (TEA). TEA using the segmented package. To identify potential inflection points in the nonlinear relationships between IR surrogate indexes and CKD risk observed in the RCS analysis, we employed segmented (piecewise) Cox regression models using the segmented package in R (version 4.5.1). Predictive performance was evaluated through receiver operating characteristic (ROC) curve analysis, including area under the curve (AUC), optimal cutoff determination, Youden’s index, sensitivity, specificity, positive predictive value (PPV), and negative predictive value (NPV). Additionally, we evaluated model discrimination via continuous net reclassification improvement (cNRI) and integrated discrimination improvement (IDI). *P* < 0.05 represented statistical significance. Statistical analyses were performed using R 4.5.1, GraphPad Prism 9.0.0, and SPSS 25.0.

## Results

### Baseline characteristics

The participant selection process is detailed in Fig. 1, with 3,403 subjects ultimately enrolled in the analysis (2,834 non-CKD and 569 CKD participants). Participants were followed for 8.4 ± 1.7 years on average. Table 1 shows the comparison of baseline characteristics. Older and female individuals implied significantly higher risks of developing CKD compared to younger and male individuals (*P* < 0.05). Individuals with CKD revealed a higher prevalence of smoking and alcohol intake, inadequate blood pressure control, poorer metabolic management, and more frequent cardiovascular comorbidities than their non-CKD counterparts. Biochemically, the CKD group revealed significantly higher levels of C-reactive protein (CRP), glycemic parameters (FPG and HbA1c), TG, serum creatinine (Scr), serum uric acid (SUA), and BUN, along with lower HDL-C and eGFR values (all *P* < 0.05). Notably, all IR markers except eGDR were significantly elevated in the CKD cohort (*P* < 0.05), with eGDR conversely showing significantly lower levels.

**Table 1:** Baseline characteristics of participants in the study

Variable	Total (*n* = 3403)	Non-CKD (*n* = 2834)	CKD (*n* = 569)	*P* value
Age (years)	57.0 (51.0, 63.0)	56.5 (50.0, 62.0)	59.0 (53.0, 66.0)	< 0.001*
Sex (male, %)	2019.0 (59.3)	1788.0 (63.1)	231.0 (40.6)	< 0.001*
Education level, n (%)				0.418
Below primary school	1653.0 (48.6)	1393.0 (49.2)	260.0 (45.7)	
Primary school	722.0 (21.2)	589.0 (20.8)	133.0 (23.4)	
Middle school	676.0 (19.9)	559.0 (19.7)	117.0 (20.6)	
High school or above	352.0 (10.3)	293.0 (10.3)	59.0 (10.4)	
Marital status (married, %)	349.0 (10.3)	289.0 (10.2)	60.0 (10.5)	0.803
Smoking status, n (%)				< 0.001*
Never	2192.0 (64.4)	1909.0 (67.4)	283.0 (49.7)	
Former	215.0 (6.3)	148.0 (5.2)	67.0 (11.8)	
Current	996.0 (29.3)	777.0 (27.4)	219.0 (38.5)	
Drinking status, n (%)				< 0.001
Never	2080.0 (61.1)	1794 (63.3)	286 (50.3)	
Former	239.0 (7.0)	186.0 (6.6)	53.0 (9.3)	
Current	1084.0 (31.9)	854.0 (30.1)	230 (40.4)	
Hypertension, n (%)	1492.0 (43.8)	1169.0 (41.3)	323.0 (56.8)	< 0.001
Heart diseases, n (%)	340.0 (10.0)	246.0 (8.7)	94.0 (16.5)	< 0.001*
Stroke diseases, n (%)	65.0 (1.9)	49.0 (1.7)	16.0 (2.8)	0.085
SBP (mmHg)	124.0 (111.5, 139.0)	123.0 (111.0, 138.0)	129.5 (115.5, 142.5)	< 0.001*
DBP (mmHg)	74.0 (66.0, 82.3)	73.5 (66.0, 81.5)	76.5 (67.5, 85.5)	< 0.001*
BMI (kg/m^2^)	23.1 (20.9, 25.7)	23.0 (20.9, 25.5)	23.6 (21.2, 26.5)	0.002*
WC (cm)	84.0 (77.2, 91.2)	83.6 (77.0, 90.8)	86.0 (78.3, 94.4)	< 0.001*
WBC (10^9/L)	5.9 (4.9, 7.1)	5.9 (4.9, 7.1)	6.0 (5.0, 7.2)	0.089
Hb (g/dL)	14.1 (12.9, 15.4)	14.05 (12.8, 15.3)	14.4 (13.1, 15.7)	< 0.001*
PLT (10^9/L)	209.0 (163.0, 258.0)	209.0 (164.0, 258.0)	208.0 (159.0, 254.0)	0.184
CRP (mg/L)	0.9 (0.5, 1.9)	0.9 (0.5, 1.8)	1.2 (0.6, 2.4)	< 0.001^*^
FPG (mg/dL)	95.6 (90.4, 99.9)	95.4 (90.2, 99.5)	96.7 (91.4, 130.1)	< 0.001^*^
HbA1c (%)	5.1 (4.9, 5.4)	5.1 (4.9, 5.4)	5.2 (4.9, 5.5)	< 0.001^*^
TC (mg/dL)	189.4 (166.2, 213.2)	189.1 (166.2, 212.5)	189.8 (165.1, 215.7)	0.594
TG (mg/dL)	100.9 (72.6, 145.1)	100.0 (71.7, 141.6)	108.8 (76.1, 161.1)	< 0.001^*^
HDL-C (mg/dL)	49.9 (41.4, 59.9)	50.3 (41.8, 60.3)	48.3 (38.7, 58.4)	< 0.001^*^
LDL-C (mg/dL)	113.7 (92.8, 135.9)	113.7 (93.2, 136.1)	113.3 (90.5, 134.5)	0.353
Scr (mg/dL)	0.7 (0.6, 0.8)	0.7 (0.6, 0.8)	0.8 (0.7, 0.9)	< 0.001^*^
SUA (mg/dL)	4.1 (3.4, 4.9)	4.1 (3.4, 4.8)	4.5 (3.8, 5.3)	< 0.001^*^
BUN (mg/dL)	14.8 (12.4, 17.6)	14.7 (12.2, 17.5)	15.2 (12.9, 18.2)	0.001^*^
eGFR (mL/min/1.73m^2^)	98.4 (81.5, 108.7)	100.1 (84.7, 109.9)	86.8 (69.7, 102.2)	< 0.001^*^
TyG	8.5 (8.2, 9.0)	8.5 (8.1, 8.9)	8.6 (8.2, 9.2)	< 0.001^*^
TyG-BMI	196.3 (174.0, 226.4)	195.2 (173.3, 224.1)	204.4 (177.9, 241.7)	< 0.001^*^
METS-IR	33.8 (29.5, 39.7)	33.5 (29.4, 39.2)	35.8 (29.9, 42.7)	< 0.001^*^
eGDR	9.7 (7.5, 11.2)	10.0 (7.6, 11.2)	8.3 (7.0, 10.8)	< 0.001^*^
CVAI	87.6 (61.6, 117.2)	85.7 (60.1, 115.3)	99.0 (70.7, 129.8)	< 0.001^*^
AIP	0.3 (0.1, 0.5)	0.3 (0.1, 0.5)	0.4 (0.1, 0.6)	< 0.001^*^

Notes: The eGFR is calculated according to the CKD-EPI formula. CKD, chronic kidney disease; SBP, systolic blood pressure; DBP, diastolic blood pressure; BMI, body mass index; WC, waist circumference; WBC, white blood cell; Hb, haemoglobin; PLT, blood platelet; CRP, C-reactive protein; FPG, fasting plasma glucose; HbA1c, haemoglobin A1c; TC, total cholesterol; TG, triglycerides; HDL-C, high-density lipoprotein cholesterol; LDL-C, low-density lipoprotein cholesterol; Scr, serum creatinine; SUA, serum uric acid; BUN, blood urea nitrogen; eGFR, estimated glomerular filtration rate; TyG, triglyceride-glucose; TyG-BMI, triglyceride glucose-body mass; METS-IR, metabolic score for insulin resistance; eGDR, estimated glucose disposal rate; CVAI, Chinese visceral adiposity index; AIP, atherogenic index of plasma

As presented in Table S1, the findings remained robust after excluding participants with non-critical missing data, suggesting that the exclusion did not significantly impact the results. Table S2 presents a comparative assessment showing that all IR indexes were similarly distributed between the missing-data group and the ultimately enrolled cohort. Consequently, it can be considered that the exclusion of these individuals did not notably impact the overall outcomes of the cohort.

### Association between IR surrogate indexes and CKD risk

To further clarify the association between IR surrogate indexes and CKD risk in diabetes subjects, three stepwise adjusted Cox regression models were developed (Table 2). In unadjusted analyses (Model 1), all IR surrogate indexes displayed significant positive associations with CKD risk, except for the inverse association observed with eGDR. The consistent trend occurred in Model 2 (adjusted for age and gender). Building upon Model 2, further multivariable adjustment for sociodemographic factors (marital status, education level), lifestyle behaviors (smoking status, alcohol status), clinical comorbidities (cardiovascular diseases), and biochemical parameters (WC, hemoglobin, CRP, HbA1c, SUA, BUN, eGFR) in Model 3 revealed that all IR surrogate indexes maintained significant associations with CKD prevalence, with effect directions consistent with previous models. Continuous variable analysis revealed that for each SD increase in IR indexes, the corresponding adjusted HRs were: TyG (1.221), TyG-BMI (1.001), METS-IR (1.003), CVAI (1.002), and AIP (1.372), whereas eGDR showed a protective effect (HR = 0.914). Quartile stratification demonstrated that participants in Q4 of TyG (HR = 1.417), TyG-BMI (HR = 1.620), METS-IR (HR = 1.360), CVAI (HR = 1.743), and AIP (HR = 1.327) had significantly higher CKD risks than those in Q1, while eGDR Q4 participants exhibited reduced risk [HR (95%CI): 0.644 (0.488, 0.849)].

**Table 2:** Regression analysis of the association between non-insulin-dependent IR surrogate indexes and CKD risk

Variable	Model 1		Model 2		Model 3	
	HR (95% CI)	*P* value	HR (95% CI)	*P* value	HR (95% CI)	*P* value
TyG (continuous)	1.376 (1.236, 1.531)	< 0.001^*^	1.428 (1.286, 1.586)	< 0.001^*^	1.221 (1.071, 1.392)	0.003^*^
Q1	Ref		Ref		Ref	
Q2	1.145 (0.889, 1.475)	0.294	1.212 (0.940, 1.561)	0.137	1.189 (0.921, 1.536)	0.184
Q3	1.286 (1.005, 1.647)	0.046^*^	1.475 (1.150, 1.892)	0.002^*^	1.367 (1.059, 1.763)	0.016^*^
Q4	1.709 (1.353, 2.159)	< 0.001^*^	1.945 (1.537, 2.461)	< 0.001^*^	1.417 (1.086, 1.849)	0.010^*^
TyG-BMI (continuous)	1.001 (1.001, 1.001)	< 0.001^*^	1.001 (1.001, 1.001)	< 0.001^*^	1.001 (1.001, 1.001)	< 0.001^*^
Q1	Ref		Ref		Ref	
Q2	0.943 (0.734, 1.211)	0.644	1.077 (0.837, 1.384)	0.565	1.061 (0.820, 1.373)	0.653
Q3	1.133 (0.891, 1.442)	0.308	1.456 (1.141, 1.859)	0.003^*^	1.377 (1.058, 1.793)	0.017^*^
Q4	1.494 (1.191, 1.874)	< 0.001^*^	2.033 (1.611, 2.566)	< 0.001^*^	1.620 (1.205, 2.178)	0.001^*^
METS-IR (continuous)	1.003 (1.002, 1.005)	< 0.001^*^	1.004 (1.002, 1.005)	< 0.001^*^	1.003 (1.002, 1.005)	< 0.001^*^
Q1	Ref		Ref		Ref	
Q2	0.829 (0.643, 1.069)	0.149	0.912 (0.707, 1.176)	0.477	0.883 (0.680, 1.147)	0.351
Q3	1.127 (0.890, 1.427)	0.322	1.320 (1.040, 1.675)	0.022^*^	1.224 (0.946, 1.585)	0.124
Q4	1.434 (1.146, 1.794)	0.002^*^	1.796 (1.430, 2.256)	< 0.001^*^	1.360 (1.016, 1.821)	0.039^*^
eGDR (continuous)	0.881 (0.849, 0.913)	< 0.001^*^	0.886 (0.854, 0.919)	< 0.001^*^	0.914 (0.876, 0.954)	< 0.001^*^
Q1	Ref		Ref		Ref	
Q2	0.728 (0.590, 0.898)	0.003^*^	0.729 (0.591, 0.900)	0.003^*^	0.750 (0.607, 0.926)	0.008^*^
Q3	0.475 (0.375, 0.603)	< 0.001^*^	0.499 (0.392, 0.634)	< 0.001^*^	0.594 (0.464, 0.761)	< 0.001^*^
Q4	0.515 (0.408, 0.649)	< 0.001^*^	0.511 (0.405, 0.646)	< 0.001^*^	0.644 (0.488, 0.849)	0.002^*^
CVAI (continuous)	1.002 (1.002, 1.003)	< 0.001^*^	1.002 (1.001, 1.003)	< 0.001^*^	1.002 (1.001, 1.002)	< 0.001^*^
Q1	Ref		Ref		Ref	
Q2	1.430 (1.101, 1.858)	0.007^*^	1.138 (0.874, 1.483)	0.337	1.196 (0.892, 1.602)	0.231
Q3	1.618 (1.252, 2.091)	< 0.001^*^	1.314 (1.012, 1.705)	0.040^*^	1.406 (1.016, 1.946)	0.040^*^
Q4	2.061 (1.612, 2.635)	< 0.001^*^	1.851 (1.440, 2.378)	< 0.001^*^	1.743 (1.174, 2.587)	0.006^*^
AIP (continuous)	1.687 (1.329, 2.142)	< 0.001^*^	1.860 (1.468, 2.356)	< 0.001^*^	1.372 (1.051, 1.791)	0.020^*^
Q1	Ref		Ref		Ref	
Q2	1.192 (0.930, 1.528)	0.165	1.254 (0.978, 1.608)	0.074	1.216 (0.945, 1.565)	0.129
Q3	1.248 (0.976, 1.597)	0.077	1.382 (1.079, 1.769)	0.010^*^	1.242 (0.961, 1.604)	0.098
Q4	1.581 (1.250, 1.999)	< 0.001^*^	1.737 (1.372, 2.199)	< 0.001^*^	1.327 (1.024, 1.720)	0.033^*^

Notes: Model 1: Unadjusted. Model 2: Adjusted for age and gender. Model 3 was adjusted for Model 2 + marital status, heart diseases, drinking status, smoking status, education level, WC, Hb, CRP, HbA1c, SUA, BUN, eGFR

IR, insulin resistance; CKD, chronic kidney disease; HR, hazard ratio; CI, confidence interval; WC, waist circumference; Hb, haemoglobin; CRP, C-reactive protein; HbA1c, haemoglobin A1c; SUA, serum uric acid; BUN, blood urea nitrogen; eGFR, estimated glomerular filtration rate; TyG, triglyceride-glucose; TyG-BMI, triglyceride glucose-body mass; METS-IR, metabolic score for insulin resistance; eGDR, estimated glucose disposal rate; CVAI, Chinese visceral adiposity index; AIP, atherogenic index of plasma

To facilitate direct comparison of the associations between various IR indices and CKD risk, we standardized the six IR indices by converting them to Z-scores and subsequently performed Cox regression analysis (Table S3). All indices demonstrated statistically significant associations with CKD risk (all *P* < 0.05). Notably, eGDR emerged as the sole protective factor, with each 1-SD increase corresponding to an approximately 20% reduction in CKD risk. Among the indices positively associated with CKD risk, TyG exhibited the strongest effect, where each 1-SD increment was associated with an approximate 17% increase in risk.

**Fig. 2:**
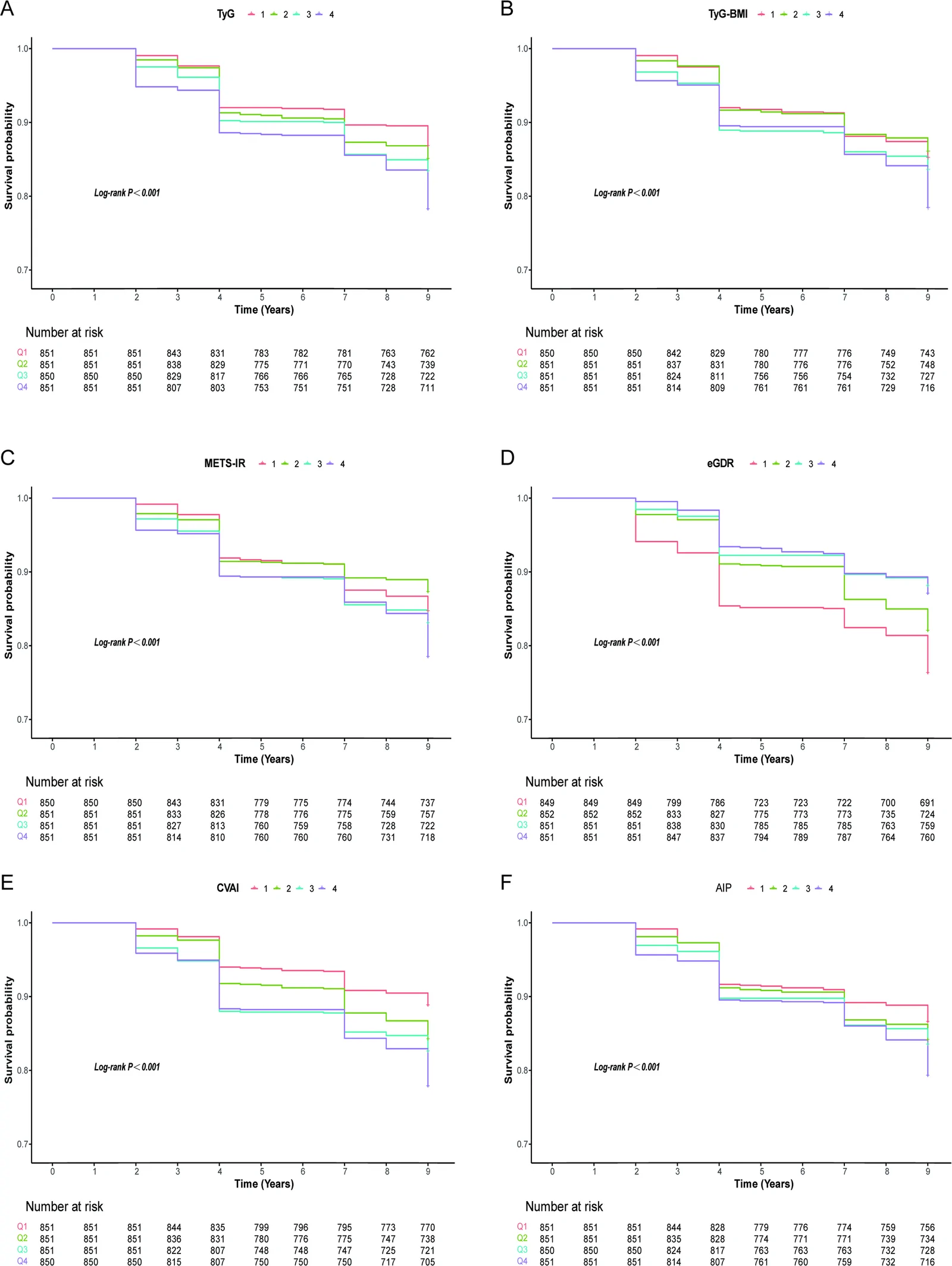
K-M curves by the category of non-insulin-dependent IR surrogate indexes. Notes: (**A**) TyG, (**B**) TyG-BMI, (**C**) METS-IR, (**D**) eGDR, (**E**) CVAI, (**F**) AIP. K-M, Kaplan-Meier; IR, insulin resistance; TyG, triglyceride-glucose; TyG-BMI, triglyceride glucose-body mass; METS-IR, metabolic score for insulin resistance; eGDR, estimated glucose disposal rate; CVAI, Chinese visceral adiposity index; AIP, atherogenic index of plasma

The K-M curves show the association between individual IR surrogate markers and cumulative CKD risk, and demonstrates progressively higher CKD event rates with increasing levels of all IR surrogates except eGDR (Fig. 2). These findings align with the Cox regression results.

**Fig. 3:**
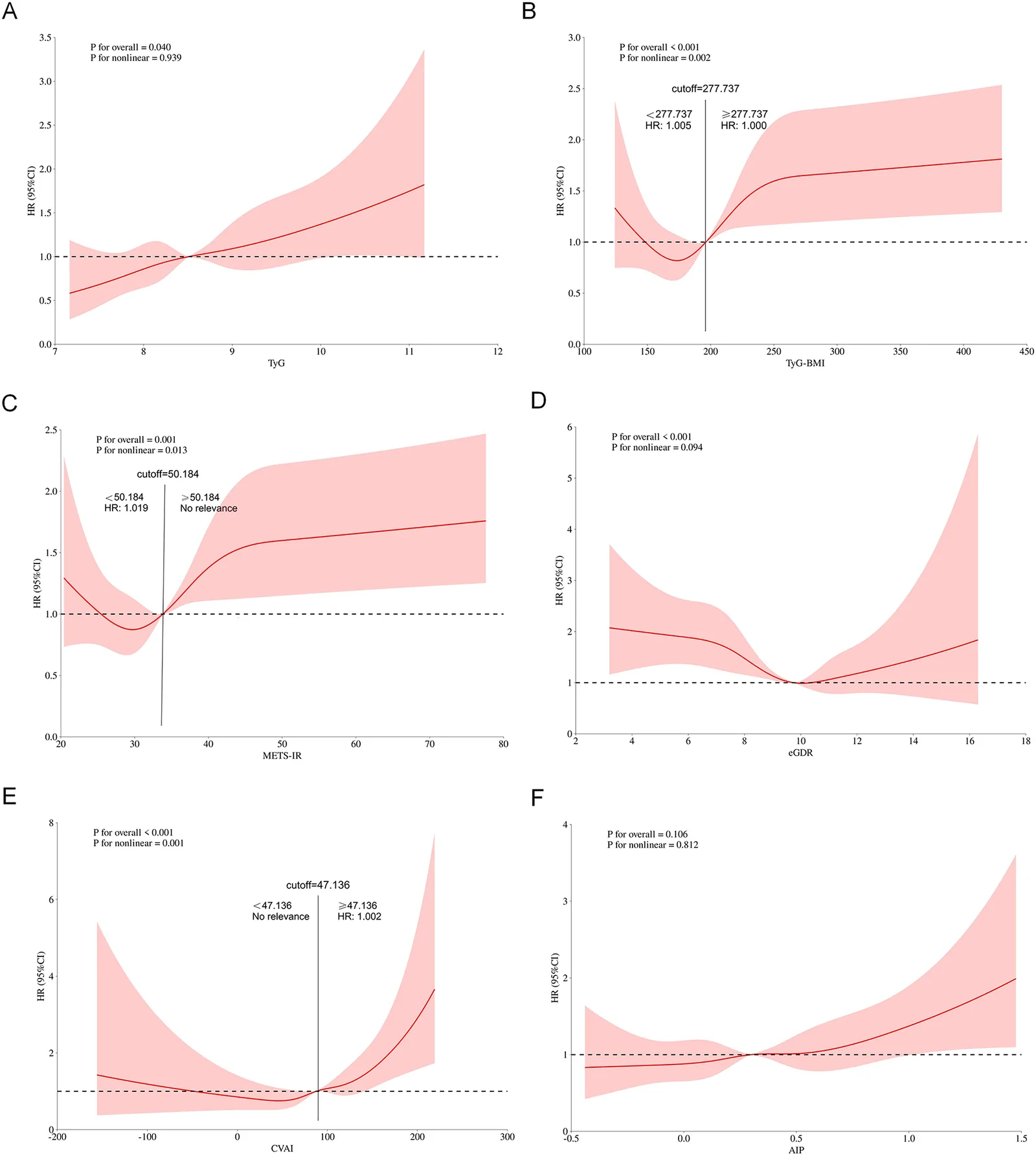
RCS for the relationships between non-insulin-dependent IR surrogate indexes and CKD risk. Notes: (**A**) TyG, (**B**) TyG-BMI, (**C**) METS-IR, (**D**) eGDR, (**E**) CVAI, (**F**) AIP. All analysis results were based on Model 3. RCS, restricted cubic spline; IR, insulin resistance; CKD, chronic kidney disease; HR, hazard ratio; CI, confidence interval; TyG, triglyceride-glucose; TyG-BMI, triglyceride glucose-body mass; METS-IR, metabolic score for insulin resistance; eGDR, estimated glucose disposal rate; CVAI, Chinese visceral adiposity index; AIP, atherogenic index of plasma

Furthermore, we assessed the relationship of all IR alternative markers with CKD using RCS and TEA (Fig. 3 and Table S4). After adjustment for all confounders, TyG and AIP exhibited a positive linear association with CKD risk (*P*_*nonlinear*_ > 0.05, Fig. 3A, F), with no evidence of a threshold effect. However, TyG-BMI and METS-IR demonstrated a nonlinear association with CKD risk (*P*_*nonlinear*_ < 0.05, Fig. 3B, C). The TEA revealed that the threshold for TyG-BMI was 277.737, below which it was positively associated with CKD risk [HR (95% CI): 1.005 (1.002, 1.009)]. Similarly, METS-IR showed a positive association with CKD risk [HR (95% CI): 1.019 (1.002, 1.036)] at values below 50.184. RCS showed a U-shaped relationship between eGDR, CVAI, and CKD risk (*P*_*nonlinear*_ < 0.05, Fig. 3D, E), but there was no threshold effect between eGDR and CKD risk. Notably, CVAI also showed a positive association with CKD risk at CVAI ≥ 47.136 [HR (95% CI): 1.002 (1.001, 1.002)].

### Subgroup analysis

**Fig. 4:**
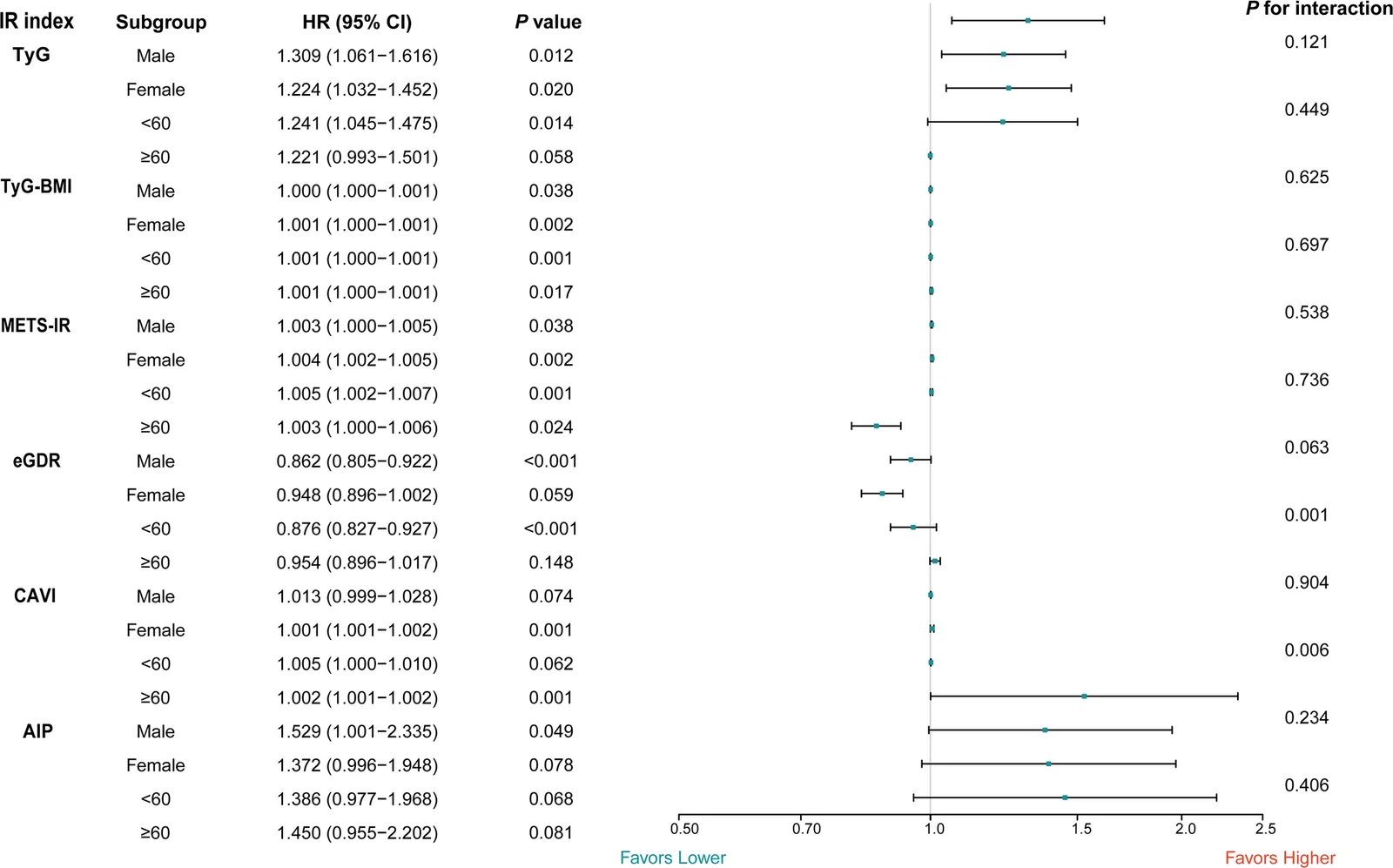
Subgroup analysis of non-insulin-dependent IR surrogate indexes (per 1 SD) for CKD risk. Notes: Subgroup analysis was based on Model 3, except for stratification variables. IR, insulin resistance; SD, standard deviation; CKD, chronic kidney disease; TyG, triglyceride-glucose; TyG-BMI, triglyceride-glucose index with body mass index; METS-IR, metabolic score for insulin resistance; eGDR, estimated glucose disposal rate; CVAI, Chinese visceral adiposity index; AIP, atherogenic index of plasma; HR, hazard ratio; CI, confidence interval

Stratification by both age and gender was employed to investigate potential variations in the associations of IR markers with CKD risk. As shown in the subgroup forest diagram (Fig. 4), TyG-BMI and METS-IR maintained significant CKD risk associations across all age and gender strata (*P* < 0.05). The eGDR demonstrated significant associations primarily in male and middle-aged participants, while CVAI showed stronger associations in female and elderly participants. Furthermore, significant interaction effects were observed between age and both eGDR (*P-interaction* = 0.001) and CVAI (*P-interaction* = 0.006).

### CKD risk prediction performance for IR surrogate indexes

**Fig. 5:**
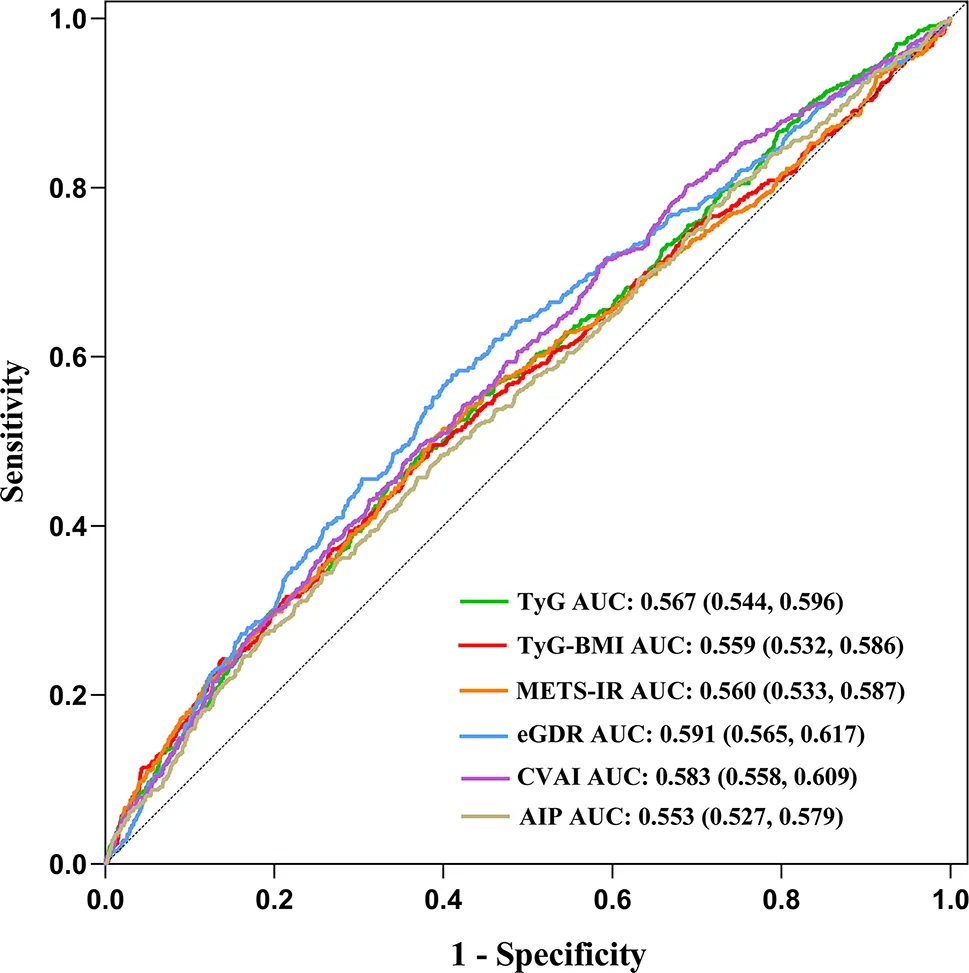
ROC curves of non-insulin-dependent IR surrogate indexes for predicting CKD. Notes: ROC, receiver operating characteristics; IR, insulin resistance; CKD, chronic kidney disease; AUC, area under the ROC curve; TyG, triglyceride-glucose; TyG-BMI, triglyceride-glucose index with body mass index; METS-IR, metabolic score for insulin resistance; eGDR, estimated glucose disposal rate; CVAI, Chinese visceral adiposity index; AIP, atherogenic index of plasma

ROC curve analysis of non-insulin-based IR surrogate indexes demonstrated moderate predictive performance for CKD risk in diabetic participants. Comparative analysis revealed eGDR demonstrated superior discriminative performance [AUC (95% CI): 0.591 (0.565–0.617)] among all markers, and significantly outperformed AIP [AUC (95% CI): 0.553 (0.527–0.579)] (*P* < 0.05, Fig. 5). Notably, eGDR also outperformed other surrogates in reclassification metrics, with a cNRI of 0.317 and an IDI of 0.014. TyG-BMI and METS-IR yielded significantly lower values compared to eGDR (*P* < 0.05, as shown in Table 3). Furthermore, based on the cutoff value, we further analyzed the association between CKD risk and IR alternative markers (Table S5). The results aligned with our prior findings: while CKD risk decreased with higher eGDR levels, all other IR markers demonstrated a positive correlation with CKD risk.

**Table 3:** Comparison of ROC curves for non-insulin-dependent IR surrogate indexes in assessing CKD risk

Variables	Sensitivity	Specificity	PPV	NPV	Cut-off value	Youden index
eGDR	0.589	0.578	0.875	0.218	8.880	0.167
TyG	0.665	0.450	0.858	0.212	8.739	0.115
TyG-BMI	0.614	0.494	0.858	0.204	206.300	0.104
METS-IR	0.615	0.503	0.861	0.207	35.746	0.118
CVAI	0.409	0.715	0.878	0.195	76.538	0.124
AIP	0.631	0.457	0.853	0.199	0.394	0.088

**Variables**	**AUC (95%CI)**	***P*** **value**	**cNRI**	***P*** **value**	**IDI**	***P*** **value**
eGDR	0.591 (0.565, 0.617)	Ref	0.317	Ref	0.014	Ref
TyG	0.567 (0.544, 0.596)	0.184	0.226	0.107	0.011	0.195
TyG-BMI	0.559 (0.532, 0.586)	0.020^*^	0.207	< 0.001^*^	0.004	< 0.001^*^
METS-IR	0.560 (0.533, 0.587)	0.031^*^	0.210	< 0.001^*^	0.005	< 0.001^*^
CVAI	0.583 (0.558, 0.609)	0.563	0.227	< 0.001^*^	0.012	0.302
AIP	0.553 (0.527, 0.579)	0.020^*^	0.161	< 0.001^*^	0.006	< 0.001^*^

Notes: ROC, receiver operating characteristics; IR, insulin resistance; CKD, chronic kidney disease; PPV, positive predictive value; NPV, negative predictive value; AUC, area under the ROC curve; cNRI, continuous net reclassification improvement; IDI, integrated discrimination improvement; eGDR, estimated glucose disposal rate; TyG, triglyceride-glucose; TyG-BMI, triglyceride glucose-body mass; METS-IR, metabolic score for insulin resistance; CVAI, Chinese visceral adiposity index; AIP, atherogenic index of plasma

## Discussion

CKD has emerged as a major global health concern, with diabetes now recognized as the leading cause of CKD worldwide [22]. IR serves as the shared pathological link between type 2 diabetes mellitus (T2DM) and diabetic kidney disease (DKD), mediating renal injury through multiple mechanisms including glucolipid metabolism dysregulation, oxidative stress, chronic inflammation, and renal fibrosis promotion, all of which ultimately contribute to CKD progression and even drive the development of end-stage renal disease (ESRD) [23–25]. Therefore, establishing practical and convenient IR assessment methods is critical for early CKD identification and timely intervention in diabetic populations. IR is characterized by metabolic dysregulation, including abnormal glucolipid metabolism, obesity, and hypertension. The IR alternative markers evaluated in this study were developed based on these clinical features, incorporating routinely available parameters such as BMI, blood pressure, and biochemical markers to provide a convenient yet effective means of assessing IR severity [26]. The current analysis employed CHARLS cohort data to assess six non-insulin-dependent IR surrogate indexes as markers of CKD risk in diabetic individuals. The 9-year large-scale prospective cohort analysis revealed the following findings: (1) all non-insulin-dependent IR surrogate indexes demonstrated independent association with CKD risk in diabetic individuals; (2) all non-insulin-dependent IR surrogate indexes showed statistically significant associations with CKD risk, though their predictive accuracy was modest; (3) eGDR demonstrated relatively better predictive performance for CKD risk in diabetic individuals.

Previous retrospective observational studies support the role of TyG index, TyG-BMI, and METS-IR as reliable markers of cardiovascular and renal complications in diabetes [16,27–29]. Specifically, *Yan* et al. [30] reported substantially elevated DKD risk at the level of Q3 of the TyG index compared with Q1 [OR (95% CI): 1.860 (1.420, 2.450)]. The TyG index, combining lipid and glucose parameters, and its BMI-incorporated derivative TyG-BMI serve as reliable IR markers, with multiple studies confirming their positive correlation with kidney disease risk in diabetes [31–34]. Supporting evidence includes a cross-sectional study of 1,080 T2DM patients identifying an independent association of TyG-BMI with renal injury, particularly CKD [OR (95% CI): 1.005 (1.001, 1.008)] [34]. In addtion, *Wu* et al.‘s [35] analysis of the United States population datasets identified the TyG index to be the most significant marker of increased CKD risk [OR (95% CI): 1.420 (1.110, 1.82)]. Our findings corroborate these observations, demonstrating that both TyG and TyG-BMI demonstrated significantly higher CKD risk in Q4 level than that in Q1, with consistent results in continuous-variable analyses. RCS analysis revealed a nonlinear relationship between TyG-BMI and CKD risk, meanwhile TEA analysis indicated positive associations within specific ranges. Regarding METS-IR, this novel composite metric integrating lipid profiles and BMI facilitates early detection of glucose metabolism disorders and obesity-related metabolic disorders [36, 37]. Furthermore, METS-IR demonstrates potential for early kidney disease screening, with studies revealing a progressive decline in eGFR levels corresponding to increasing METS-IR values, indicating a significant inverse correlation [38]. Notably, our Cox regression analysis revealed a significantly increased CKD risk at the METS-IR Q4 level compared to Q1 [HR (95% CI): 1.360 (1.016, 1.821)]. Subgroup analyses demonstrated consistent positive associations between METS-IR and DKD risk each age and gender stratification, and TEA confirmed this positive relationship. Regarding predictive performance, METS-IR showed modest predictive ability for DKD risk among diabetic individuals, consistent with its significant but weak discriminative capacity observed in ROC analysis. For TyG-BMI and METS-IR, the threshold effects suggest that beyond certain levels, further increases may not proportionally elevate risk, possibly reflecting a saturation effect where maximal IR-related renal injury has already been reached [16]. Alternatively, these inflection points may represent the transition from compensatory metabolic adaptation to decompensated organ damage.

The eGDR integrates blood glucose, blood lipid profiles, blood pressure, and body composition measurements to predict tissue glucose uptake and utilization efficiency per unit time [39]. This index has been primarily employed in IR studies focusing on diabetes and metabolic disorders. As eGDR exhibits an inverse relationship with IR severity, numerous studies have demonstrated that lower eGDR values significantly correlate with increased risks of cardiovascular and cerebrovascular events in diabetic populations [9, 40]. Notably, studies in type 1 diabetes mellitus (T1DM) patients demonstrated a significant inverse association between eGDR and microvascular complications, especially retinopathy and kidney disease in young individuals [6], supporting its utility in risk assessment for glucose metabolism disorder-related microvascular outcomes. The present investigation was designed to characterize both the epidemiological association and prognostic utility of eGDR for CKD risk stratification in individuals with diabetes. Given that the primary CKD risk factors in diabetes, including glycemic control, lipid levels, blood pressure, and body composition, are all incorporated into the eGDR calculation, we hypothesized a strong association between eGDR and CKD risk in diabetic individuals. Our Cox regression analyses confirmed this, demonstrating significant inverse associations between eGDR and CKD risk across both continuous and categorical variable in all Models, regardless of covariate adjustments. Although the RCS suggested nonlinearity, no significant threshold was identified in segmented regression, indicating that the protective association operates continuously across the observed range. This finding underscores the utility of eGDR as a consistent risk marker without evident ceiling effects [41]. Subgroup analyses further revealed higher eGDR levels among males and younger individuals, coupled with a significant gender-by-eGDR interaction in relation to CKD risk. K-M curves substantiated these findings, showing decreasing cumulative CKD risk with progressively higher eGDR levels. Furthermore, ROC analysis, along with cNRI and IDI, indicated that eGDR had relatively better predictive performance compared to the other non-insulin-dependent IR surrogate markers, although the absolute discriminative ability of all indices remained modest. These results align with findings from an NHANES-based cross-sectional study that reported eGDR as having the strongest association with eGFR among traditional IR indexes, establishing it as the optimal CKD risk marker [41]. Compared with this study, our research specifically investigated the correlation between CKD risk and eGDR in diabetic participants, with particular emphasis on analyzing the role of eGDR in CKD risk among Chinese middle-aged and elderly diabetes. This approach enables early identification and intervention for CKD risk in this population. A methodological caveat warrants consideration: eGDR incorporates hypertension and HbA1c, notably, both are established CKD risk factors. Other IR surrogates, in contrast, are derived solely from metabolic parameters. Therefore, eGDR’s superior predictive performance may partly stem from this compositional advantage, not necessarily greater accuracy in reflecting IR itself. For CKD risk prediction, this supports eGDR’s utility as a composite marker. However, for studies aiming to isolate the pathological mechanism of IR, purer metabolic indices are likely more appropriate.

CVAI is an index developed for assessing visceral fat distribution and metabolic risk in the Chinese population [42]. CVAI shows significant associations with diabetes, obesity, cardiovascular disease, stroke and atherosclerosis [43], and demonstrates better suitability for evaluating metabolic characteristics in Chinese individuals, compared to western visceral fat indices (VAI). RCS analysis revealed a U-shaped correlation between CVAI and CKD risk, further supported by K-M curves, whereas TEA indicated a positive association. Moderate visceral adiposity may confer protective metabolic buffering, whereas excessive accumulation promotes pathogenic adipokine secretion, systemic inflammation, and renal fibrosis [44]. Similar to eGDR, subgroup analyses demonstrated an interaction between CVAI and gender in predicting CKD risk. Additionally, CVAI showed modest predictive performance for CKD risk among diabetic individuals, with AUC, cNRI and IDI values second only to eGDR, though still reflecting limited discriminative ability. An intriguing finding of our study is the significant interaction between age and both eGDR (*P-interaction* = 0.001) and CVAI (*P-interaction* = 0.006) in relation to CKD risk. These interactions suggest that the associations of these specific IR surrogates with kidney outcomes are not uniform across the lifespan, warranting a deeper exploration of the underlying biological and clinical factors. The age-modified effect of eGDR may reflect cumulative physiological changes with aging. eGDR incorporates WC, hypertension, and HbA1c, factors that tend to worsen with age due to increased central adiposity, vascular stiffening, and longer diabetes duration [45]. This cumulative burden may attenuate the protective effect of higher eGDR in older adults. Our subgroup analysis supports this, showing a stronger protective association in middle-aged participants, suggesting that earlier intervention on eGDR components (e.g., weight and blood pressure control) may offer greater renal benefits. Conversely, the stronger CVAI-CKD association in elderly and female participants aligns with age-related visceral fat redistribution, particularly postmenopausal [44]. Visceral adipose tissue promotes inflammation and oxidative stress, contributing to renal fibrosis [46]. Clinically, these findings suggest age-specific risk stratification: CVAI may be more valuable in the elderly for capturing visceral adiposity burden, while eGDR may better identify middle-aged individuals at early risk when lifestyle interventions are most effective.

AIP was originally developed to assess plasma atherogenicity risk but has since been widely studied in relation to IR and cardiovascular disease [19]. Meta-analytical studies [47] have identified that elevated AIP, reflecting increased plasma atherogenicity, regards as a reliable marker for coronary artery disease risk beyond conventional risk factors. While *Wang* et al. [48] found no significant association between CKD and AIP but suggested a nonlinear relationship, another NHANES study clearly demonstrated that elevated AIP levels increase CKD risk [49]. Thus, the role of AIP in predicting CKD risk in diabetic individuals remains inconclusive. Our research identified a positive linear relationship between AIP and CKD, with Cox analysis confirming that elevated AIP increases CKD risk in the diabetic individuals. This discrepancy may reflect our diabetic cohort, wherein pre-existing metabolic disturbances amplify the atherogenic effect on renal microvasculature, eliminating any ‘protective’ lower range [19]. However, the predictive performance of AIP for CKD risk was the weakest among all non-insulin-dependent IR surrogate indexes, consistent with its lowest AUC value. Overall, this study clearly establishes a significant association between AIP and CKD risk.

A further layer of complexity warrants consideration: the bidirectional relationship between diabetes and CKD. While our study focused on IR predicting CKD, kidney dysfunction can also perturb glucose homeostasis. CKD, particularly after acute kidney injury, increases risks of hypoglycemia and hyperglycemic crises due to impaired insulin clearance, reduced renal gluconeogenesis, and altered drug pharmacokinetics [50–52]. Additionally, patients presenting with hyperglycemic crisis at diabetes onset have higher subsequent CKD risk [53]. This bidirectional interplay suggests a vicious cycle wherein diabetes promotes kidney injury, which in turn exacerbates glycemic dysregulation. Future studies should incorporate dynamic assessments of both glycemic control and renal function.

This study is among the first to comprehensively compare the predictive capabilities of various non-insulin-dependent IR markers for CKD risk in diabetic populations, offering important clinical insights into optimal risk stratification tools. The prospective cohort design utilizing CHARLS data, with long follow-up periods, significantly strengthens the causal inferences that can be drawn from our findings. Methodologically, we employed robust statistical approaches including multivariate Cox regression, K-M survival analysis, subgroup stratification, and comparative evaluation of multiple predictive performance metrics to thoroughly characterize each index. Several limitations should be acknowledged. First, the temporal precision of CKD event ascertainment may be insufficient, potentially obscuring true survival risk patterns. Future studies could improve this by recording follow-up intervals in months for more precise risk assessment. As a consequence of the global disruption caused by the COVID-19 pandemic, blood biomarker data from the final wave of the CHARLS database were unavailable, introducing potential bias in the ascertainment of CKD outcomes. Second, our CKD determination relied solely on eGFR < 60 mL/min/1.73 m² and self-reported data without incorporating UACR, meaning our findings primarily apply to moderate-to-advanced CKD (stages G3-G5). This may lead to underdetection of early-stage cases and affect prevalence estimates. Incorporating UACR, comprehensive biochemical profiling, and active participant follow-up would enhance endpoint accuracy. Third, data on chronic inflammatory conditions (e.g., chronic hepatitis, autoimmune diseases) were unavailable in CHARLS. These conditions may influence both IR and CKD risk, and their absence from adjustment models represents a potential source of residual confounding. Finally, while our findings are robust for middle-aged and elderly Chinese diabetic population, generalizability to other ethnicities and healthcare settings requires further validation through international studies.

## Conclusion

Our findings demonstrate that all non-insulin-dependent IR surrogate indexes exhibit significant associations with CKD risk in Chinese middle-aged and elderly diabetic populations, although their predictive accuracy is modest. Among these markers, eGDR showed relatively better performance for CKD risk assessment. However, given the weak discriminative ability observed, these indices should be used as complementary tools rather than standalone predictors in clinical practice. Nevertheless, multinational validation studies incorporating ethnically diverse populations are warranted to corroborate these findings and strengthen their clinical applicability across varying genetic and environmental backgrounds.

## Supplementary Information

Below is the link to the electronic supplementary material.

Supplementary Material 1

## Data Availability

No datasets were generated or analysed during the current study. Clinical trial number: not applicable.
