# Supplementary material for: Non-insulin-dependent insulin resistance surrogate indexes for predicting chronic kidney disease in diabetic individuals: a prospective cohort study
Source: BMC Endocr Disord. 2026 Jun 26;26:247. doi: 10.1186/s12902-026-02362-3 (PMC13505027; doi:10.1186/s12902-026-02362-3)
Supplement: Supplementary file 1 [file 12902_2026_2362_MOESM1_ESM.docx]

Table S1. Sensitivity analysis excluding participants with missing data for non-critical variables (N=3333).

| **Variable** | **Excluding participants with missing data（n=3333）** | **Final analytic cohort（n=3403）** | ***P* value** |
| --- | --- | --- | --- |
| Age (years) | 57.0 (51.0, 63.0) | 57.0 (51.0, 63.0) | 0.925 |
| Sex (male, %) | 1986.0 (59.6) | 2019.0 (59.3) | 0.831 |
| Education level, n (%) |  |  | 1.000 |
| Below primary school | 1618.0 (48.5) | 1653.0 (48.6) |  |
| Primary school | 710.0 (21.3) | 722.0 (21.2) |  |
| Middle school | 662.0 (19.9) | 676.0 (19.9) |  |
| High school or above | 343.0 (10.3) | 352.0 (10.3) |  |
| Marital status (married, %) | 333.0 (10.0) | 349.0 (10.3) | 0.719 |
| Smoking status, n (%) |  |  | 0.998 |
| Never | 2149.0 (64.5) | 2193.0 (64.4) |  |
| Former | 210.0 (6.3) | 215.0 (6.3) |  |
| Current | 974.0 (29.2) | 996.0 (29.3) |  |
| Drinking status, n (%) |  |  | 0.982 |
| Never | 2040.0 (61.2) | 2080.0 (61.1) |  |
| Former | 237.0 (7.1) | 240.0 (7.1) |  |
| Current | 1056.0 (31.7) | 1084.0 (31.8) |  |
| Hypertension, n (%) | 1469.0 (44.1) | 1493.0 (43.9) | 0.849 |
| Heart diseases, n (%) | 338.0 (10.1) | 340.0 (10.0) | 0.838 |
| Stroke diseases, n (%) | 63.0 (1.9) | 65.0 (1.9) | 0.952 |
| SBP (mmHg) | 124.0 (111.5, 139.0) | 124.0 (111.5, 139.0) | 0.938 |
| DBP (mmHg) | 74.0 (66.0, 82.0) | 74.0 (66.0, 82.3) | 0.917 |
| BMI (kg/m^2^) | 23.0 (20.9, 25.7) | 23.1 (20.9, 25.7) | 0.926 |
| WC (cm) | 84.0 (77.2, 91.3) | 84.0 (77.2, 91.2) | 0.934 |
| WBC (10^9/L) | 5.9 (4.9, 7.1) | 5.9 (4.9, 7.1) | 0.973 |
| Hb (g/dL) | 14.1 (12.9, 15.4) | 14.1 (12.9, 15.4) | 0.977 |

Table S1. (continued)

| **Variable** | **Excluding participants with missing data（n=3333）** | **Final analytic cohort（n=3403）** | ***P* value** |
| --- | --- | --- | --- |
| PLT (10^9/L) | 209.0 (163.0, 258.0) | 209.0 (163.0, 258.0) | 0.931 |
| CRP (mg/L) | 0.9 (0.5, 1.9) | 0.9 (0.5, 1.9) | 0.905 |
| FPG (mg/dL) | 95.6 (90.4, 99.9) | 95.6 (90.4, 99.9) | 0.963 |
| HbA1c (%) | 5.1 (4.9, 5.4) | 5.1 (4.9, 5.4) | 0.905 |
| TC (mg/dL) | 189.4 (166.2, 213.4) | 189.4 (166.2, 213.4) | 0.969 |
| TG (mg/dL) | 100.9 (72.6, 145.1) | 100.9 (72.6, 145.1) | 0.994 |
| HDL-C (mg/dL) | 49.9 (41.4, 59.9) | 49.9 (41.4, 59.9) | 0.999 |
| LDL-C (mg/dL) | 113.7 (92.8, 136.1) | 113.7 (92.8, 135.9) | 0.992 |
| Scr (mg/dL) | 0.7 (0.6, 0.8) | 0.7 (0.6, 0.8) | 0.836 |
| SUA (mg/dL) | 4.1 (3.5, 4.9) | 4.1 (3.5, 4.9) | 0.826 |
| BUN (mg/dL) | 14.8 (12.4, 17.6) | 14.8 (12.4, 17.6) | 0.987 |
| eGFR (mL/min/1.73m^2^) | 98.4 (81.5, 108.7) | 98.4 (81.5, 108.7) | 0.912 |
| TyG index | 8.5 (8.2, 9.0) | 8.5 (8.2, 9.0) | 0.995 |
| TyG-BMI index | 196.5 (173.8, 226.6) | 196.3 (174.0, 226.3) | 0.962 |
| TG/HDL-C | 2.0 (1.3, 3.3) | 2.0 (1.3, 3.3) | 0.994 |
| METS-IR | 33.8 (29.5, 39.8) | 33.8 (29.5, 39.7) | 0.993 |
| eGDR | 9.7 (7.5, 11.2) | 9.7 (7.5, 11.1) | 0.912 |
| CVAI | 87.6 (61.5, 117.2) | 87.6 (61.6, 117.2) | 0.960 |
| AIP | 0.3 (0.1, 0.5) | 0.3 (0.1, 0.5) | 0.994 |

Notes: The eGFR is calculated according to the CKD-EPI formula. SBP, systolic blood pressure; DBP, diastolic blood pressure; BMI, body mass index; WC, waist circumference; WBC, white blood cell; Hb, haemoglobin; PLT, blood platelet; CRP, C-reactive protein; FPG, fasting plasma glucose; HbA1c, haemoglobin A1c; TC, total cholesterol; TG, triglycerides; HDL-C, high-density lipoprotein cholesterol; LDL-C, low-density lipoprotein cholesterol; Scr, serum creatinine; SUA, serum uric acid; BUN, blood urea nitrogen; eGFR, estimated glomerular filtration rate; TyG index, triglyceride-glucose index; TyG-BMI index, triglyceride glucose-body mass index; TG/HDL-C, triglyceride to high-density lipoprotein cholesterol ratio; METS-IR, metabolic score for insulin resistance; eGDR, estimated glucose disposal rate; CVAI, Chinese visceral adiposity index; AIP, atherogenic index of plasma.

Table S2. Sensitivity analysis of participants all excluded and retained in the study

| **Variable** | **Total (n=5034)** | **Excluded（n=1631）** | **Retained（n=3403）** | ***P* value** |
| --- | --- | --- | --- | --- |
| Age (years) | 58.0 (51.0, 64.0) | 60.0 (53.0, 68.0) | 57.0 (51.0, 63.0) | <0.001 |
| Sex (male, %) | 2878.0 (57.2) | 858.0 (52.6) | 2019.0 (59.3) | <0.001 |
| Education level, n (%) |  |  |  | 0.020 |
| Below primary school | 2428.0 (48.3) | 775.0 (47.7) | 1653.0 (48.6) |  |
| Primary school | 1055.0 (21.0) | 333.0 (20.5) | 722.0 (21.2) |  |
| Middle school | 978.0 (19.5) | 302.0 (18.6) | 676.0 (19.9) |  |
| High school or above | 568.0 (11.3) | 216.0 (13.3) | 352.0 (10.3) |  |
| Marital status (married, %) | 571.0 (11.3) | 222.0 (13.6) | 349.0 (10.3) | <0.001 |
| Smoking status, n (%) |  |  |  | <0.001 |
| Never | 3115.0 (61.9) | 922.0 (56.6) | 2193.0 (64.4) |  |
| Former | 350.0 (7.0) | 135.0 (8.3) | 215.0 (6.3) |  |
| Current | 1569.0 (31.2) | 573.0 (35.2) | 996.0 (29.3) |  |
| Drinking status, n (%) |  |  |  | 0.085 |
| Never | 3041.0 (60.4) | 961.0 (59.0) | 2080.0 (61.1) |  |
| Former | 382.0 (7.6) | 142.0 (8.7) | 240.0 (7.1) |  |
| Current | 1611.0 (32.0) | 527.0 (32.3) | 1084.0 (31.8) |  |
| Hypertension, n (%) | 1851.0 (45.5) | 358.0 (53.9) | 1493.0 (43.9) | <0.001 |
| Heart diseases, n (%) | 587.0 (11.7) | 247.0 (15.2) | 340.0 (10.0) | <0.001 |
| Stroke diseases, n (%) | 141.0 (2.8) | 76.0 (4.7) | 65.0 (1.9) | <0.001 |
| SBP (mmHg) | 125.0 (112.5, 140.0) | 130.0 (117.0, 145.5) | 124.0 (111.5, 139.0) | <0.001 |
| DBP (mmHg) | 74.0 (66.0, 82.5) | 75.5 (67.0, 84.0) | 74.0 (66.0, 82.3) | 0.002 |
| BMI (kg/m^2^) | 23.0 (20.8, 25.7) | 22.7 (20.2, 25.6) | 23.1 (20.9, 25.7) | 0.010 |
| WC (cm) | 84.0 (77.2, 91.6) | 85.0 (77.2, 93.0) | 84.0 (77.2, 91.2) | 0.089 |
| WBC (10^9/L) | 5.9 (4.9, 7.1) | 5.80 (4.9, 7.1) | 5.90 (4.9, 7.1) | 0.939 |
| Hb (g/dL) | 14.2 (12.9, 15.5) | 14.3 (13.0, 15.6) | 14.1 (12.9, 15.4) | 0.004 |
| PLT (10^9/L) | 207.0 (161.0, 254.0) | 200.0 (159.0, 247.0) | 209.0 (163.0, 258.0) | <0.001 |
| CRP (mg/L) | 1.0 (0.5, 2.1) | 1.2 (0.6, 2.7) | 0.9 (0.5, 1.9) | <0.001 |

Table S2. (continued)

| **Variable** | **Total (n=5034)** | **Excluded（n=1631）** | **Retained（n=3403）** | ***P* value** |
| --- | --- | --- | --- | --- |
| FPG (mg/dL) | 95.8 (90.5, 108.0) | 96.3 (90.9, 126.5) | 95.6 (90.4, 99.9) | <0.001 |
| HbA1c (%) | 5.1 (4.9, 5.5) | 5.1 (4.9, 5.6) | 5.1 (4.9, 5.4) | 0.014 |
| TC (mg/dL) | 189.1 (165.7, 214.2) | 188.3 (164.3, 216.5) | 189.4 (166.2, 213.4) | 0.777 |
| TG (mg/dL) | 101.8 (73.5, 147.8) | 104.4 (75.2, 154.9) | 100.9 (72.6, 145.1) | 0.016 |
| HDL-C (mg/dL) | 49.5(40.6 59.9) | 48.7 (39.8, 59.9) | 49.9 (41.4, 59.9) | 0.019 |
| LDL-C (mg/dL) | 113.7 (92.4, 136.5) | 112.9 (90.9, 136.9) | 113.7 (92.8, 135.9) | 0.167 |
| Scr (mg/dL) | 0.7 (0.6, 0.9) | 0.8 (0.6, 0.9) | 0.7 (0.6, 0.8) | <0.001 |
| SUA (mg/dL) | 4.2 (3.5, 5.0) | 4.3 (3.5, 5.2) | 4.1 (3.5, 4.9) | <0.001 |
| BUN (mg/dL) | 14.9 (12.4, 17.8) | 15.0 (12.5, 18.2) | 14.8 (12.4, 17.6) | 0.038 |
| eGFR (mL/min/1.73m^2^) | 96.9 (79.8, 108.0) | 92.6 (76.7, 105.5) | 98.4 (81.5, 108.7) | <0.001 |
| TyG index | 8.5 (8.1, 9.0) | 8.5 (8.1, 9.0) | 8.5 (8.2, 9.0) | 0.187 |
| TyG-BMI index | 195.7 (173.7, 226.1) | 193.6 (172.9, 224.6) | 196.3 (174.0, 226.3) | 0.159 |
| TG/HDL-C | 2.0 (1.3, 3.3) | 2.0 (1.3, 3.2) | 2.0 (1.3, 3.3) | 0.671 |
| METS-IR | 33.8 (29.4, 39.6) | 33.8 (29.4, 39.5) | 33.8 (29.5, 39.7) | 0.605 |
| eGDR | 9.8 (7.5, 11.2) | 9.9 (7.4, 11.2) | 9.7 (7.5, 11.1) | 0.415 |
| CVAI | 86.9 (61.3, 117.1) | 85.4 (60.2, 115.8) | 87.6 (61.6, 117.2) | 0.136 |
| AIP | 0.3 (0.1, 0.5) | 0.3 (0.1, 0.5) | 0.3 (0.1, 0.5) | 0.756 |

Notes: The eGFR is calculated according to the CKD-EPI formula. SBP, systolic blood pressure; DBP, diastolic blood pressure; BMI, body mass index; WC, waist circumference; WBC, white blood cell; Hb, haemoglobin; PLT, blood platelet; CRP, C-reactive protein; FPG, fasting plasma glucose; HbA1c, haemoglobin A1c; TC, total cholesterol; TG, triglycerides; HDL-C, high-density lipoprotein cholesterol; LDL-C, low-density lipoprotein cholesterol; Scr, serum creatinine; SUA, serum uric acid; BUN, blood urea nitrogen; eGFR, estimated glomerular filtration rate; TyG index, triglyceride-glucose index; TyG-BMI index, triglyceride glucose-body mass index; TG/HDL-C, triglyceride to high-density lipoprotein cholesterol ratio; METS-IR, metabolic score for insulin resistance; eGDR, estimated glucose disposal rate; CVAI, Chinese visceral adiposity index; AIP, atherogenic index of plasma.

Table S3. Association between Z-score standardized IR surrogate indexes and CKD risk in Cox regression models

| **Variable** | **Model 1** | | **Model 2** | | **Model 3** | |
| --- | --- | --- | --- | --- | --- | --- |
|  | **HR (95% CI)** | ***P* value** | **HR (95% CI)** | ***P* value** | **HR (95% CI)** | ***P* value** |
| TyG | 1.292 (1.185, 1.408) | <0.001^*^ | 1.331 (1.219, 1.453) | <0.001^*^ | 1.173 (1.056, 1.302) | 0.003^*^ |
| TyG-BMI | 1.041 (1.041, 1.041) | <0.001^*^ | 1.041 (1.041, 1.041) | <0.001^*^ | 1.041 (1.041, 1.041) | <0.001^*^ |
| METS-IR | 1.030 (1.020, 1.051) | <0.001^*^ | 1.041 (1.020, 1.051) | <0.001^*^ | 1.030 (1.020, 1.051) | <0.001^*^ |
| eGDR | 0.730 (0.669, 0.797) | <0.001^*^ | 0.740 (0.678, 0.808) | <0.001^*^ | 0.802 (0.729, 0.882) | <0.001^*^ |
| CVAI | 1.083 (1.083, 1.127) | <0.001^*^ | 1.083 (1.041, 1.127) | <0.001^*^ | 1.083 (1.041, 1.083) | <0.001^*^ |
| AIP | 1.164 (1.093, 1.249) | <0.001^*^ | 1.201 (1.122, 1.284) | <0.001^*^ | 1.101 (1.016, 1.192) | 0.020^*^ |

Notes: Model 1: Unadjusted. Model 2: Adjusted for age and gender. Model 3 was adjusted for Model 2+marital status, heart diseases, drinking status, smoking status, education level, WC, Hb, CRP, HbA1c, SUA, BUN, eGFR.

IR, insulin resistance; CKD, chronic kidney disease; HR, hazard ratio; CI, confidence interval; WC, waist circumference; Hb, haemoglobin; CRP, C-reactive protein; HbA1c, haemoglobin A1c; SUA, serum uric acid; BUN, blood urea nitrogen; eGFR, estimated glomerular filtration rate; TyG, triglyceride-glucose; TyG-BMI, triglyceride glucose-body mass; METS-IR, metabolic score for insulin resistance; eGDR, estimated glucose disposal rate; CVAI, Chinese visceral adiposity index; AIP, atherogenic index of plasma.

Table S4. TEA of alternative IR indexes and CKD risk in diabetic participants

|  | **HR (95%CI)** | ***P* value** |
| --- | --- | --- |
| TyG |  |  |
| Total | 1.221 (1.071, 1.392) | 0.003 |
| Segmented logistic regression model |  |  |
| Inflection point | 8.177 |  |
| <8.177 | 1.672 (0.716, 3.902) | 0.235 |
| ≥8.177 | 1.175 (0.994, 1.389) | 0.058 |
| *P* for likelihood test |  | 0.752 |
| TyG-BMI |  |  |
| Total | 1.001 (1.000, 1.001) | <0.001 |
| Segmented logistic regression model |  |  |
| Inflection point | 277.737 |  |
| <277.737 | 1.005 (1.002, 1.009) | 0.002 |
| ≥277.737 | 1.000 (1.000, 1.001) | 0.130 |
| *P* for likelihood test |  | 0.003 |
| METS-IR |  |  |
| Total | 1.003 (1.002, 1.005) | <0.001 |
| Segmented logistic regression model |  |  |
| Inflection point | 50.184 |  |
| <50.184 | 1.019 (1.002, 1.036) | 0.028 |
| ≥50.184 | 1.001 (0.999, 1.004) | 0.321 |
| *P* for likelihood test |  | 0.025 |
| eGDR |  |  |
| Total | 0.914 (0.876, 0.954) | <0.001 |
| Segmented logistic regression model |  |  |
| Inflection point | 10.732 |  |
| <10.732 | 0.894 (0.843, 0.949) | <0.001 |
| ≥10.732 | 0.823 (0.601, 1.128) | 0.226 |
| *P* for likelihood test |  | 0.228 |
| CVAI |  |  |
| Total | 1.002 (1.001, 1.002) | <0.001 |
| Segmented logistic regression model |  |  |
| Inflection point | 47.136 |  |
| <47.136 | 0.999 (0.990, 1.008) | 0.850 |
| ≥47.136 | 1.002 (1.001, 1.002) | <0.001 |
| *P* for likelihood test |  | 0.020 |

Table S4. (continued)

|  | **HR (95%CI)** | ***P* value** |
| --- | --- | --- |
| AIP |  |  |
| Total | 1.372 (1.051, 1.791) | 0.020 |
| Segmented logistic regression model |  |  |
| Inflection point | 0.314 |  |
| <0.314 | 0.100 (0.006, 1.632) | 0.106 |
| ≥0.314 | 1.364 (1.023, 1.820) | 0.035 |
| *P* for likelihood test |  | 0.626 |

Notes: The model was adjusted for age and gender, marital status, heart diseases, drinking status, smoking status, education level, WC, Hb, CRP, HbA1c, SUA, BUN, eGFR.

TEA, threshold effect analysis; IR, insulin resistance; CKD, chronic kidney disease; HR, hazard ratio; CI, confidence interval; WC, waist circumference; Hb, haemoglobin; CRP, C-reactive protein; HbA1c, haemoglobin A1c; SUA, serum uric acid; BUN, blood urea nitrogen; eGFR, estimated glomerular filtration rate; TyG, triglyceride-glucose; TyG-BMI, triglyceride glucose-body mass; METS-IR, metabolic score for insulin resistance; eGDR, estimated glucose disposal rate; CVAI, Chinese visceral adiposity index; AIP, atherogenic index of plasma.

Table S5 The incidence of CKD as the escalation of non-insulin-dependent surrogate IR indexes

| **Variables** | **CKD** | **Non-CKD** | **The incidence of CKD (%)** | ***P* value** |
| --- | --- | --- | --- | --- |
| **TyG** |  |  |  | <0.001 |
| <8.739 | 314 | 1886 | 14.3 |  |
| ≥8.739 | 255 | 948 | 21.2 |  |
| **TyG-BMI** |  |  |  | <0.001 |
| <206.300 | 288 | 1741 | 14.2 |  |
| ≥206.300 | 281 | 1093 | 20.5 |  |
| **METS-IR** |  |  |  | <0.001 |
| <35.746 | 283 | 1742 | 14.0 |  |
| ≥35.746 | 286 | 1092 | 20.8 |  |
| **eGDR** |  |  |  | <0.001 |
| <8.880 | 329 | 1164 | 22.0 |  |
| ≥8.880 | 240 | 1670 | 12.5 |  |
| **CVAI** |  |  |  | <0.001 |
| <76.538 | 162 | 1160 | 12.3 |  |
| ≥76.538 | 407 | 1674 | 19.6 |  |
| **AIP** |  |  |  | <0.001 |
| <0.394 | 311 | 1788 | 14.8 |  |
| ≥0.394 | 258 | 1046 | 19.8 |  |

Notes: CKD, chronic kidney disease; IR, insulin resistance; TyG, triglyceride-glucose; TyG-BMI, triglyceride glucose-body mass; METS-IR, metabolic score for insulin resistance; eGDR, estimated glucose disposal rate; CVAI, Chinese visceral adiposity index; AIP, atherogenic index of plasma.
